# Use of instant messaging in electrophysiological clinical practice in Latin America: a LAHRS survey

**DOI:** 10.1093/europace/euac080

**Published:** 2022-06-21

**Authors:** Márcio Jansen de Oliveira Figueiredo, Alejandro Cuesta, David Duncker, Serge Boveda, Federico Guerra, Manlio F Márquez

**Affiliations:** Cardiology, Electrophysiology Service, University of Campinas (UNICAMP) Hospital, Campinas, Brazil; Cardiology Department, Hospital de Clínicas, Facultad de Medicina, Universidad de la República, Montevideo, Uruguay; Hannover Heart Rhythm Center, Department of Cardiology and Angiology, Hannover Medical School, Hannover, Germany; Heart Rhythm Management Department, Clinique Pasteur, 45 Avenue de Lombez, 31076 Toulouse, France; Department of Biomedical Sciences and Public Health, University Hospital "Umberto I-Lancisi-Salesi", Marche Polytechnic University, Ancona, Italy; Clinical Investigation Department, National Institute of Cardiology Ignacio Chavez, Mexico City, Mexico

**Keywords:** Electrophysiology, Clinical practice, Digital health

## Abstract

**Aims:**

Instant messaging (IM) platforms are a prominent component of telemedicine and a practical tool for sharing clinical data and counselling. Purpose of the survey was to inquire about the use of IM, the platforms used, frequency, recipients, and contents in Latin America region.

**Methods and results:**

An online survey was sent to medical community via newsletter and social media channels. The survey consisted in 22 questions, in Spanish and Portuguese, collected on SurveyMonkey. A total of 125 responders from 13 Latin-American countries (79% male, mean age 46.1 ± 9.7 years) completed the survey. Most of the responders declared that they send (88.8%) and receive (97.6%) clinical data through IM apps. Most senders declare that they anonymize clinical data before sending (71.0 ± 38.3%), but that the data received is anonymized only in 51.4 ± 33.5%. The most common tests shared with other physicians were 12-lead electrocardiograms (99.2%), followed by Holter recordings (68.0%) and tracings from electrophysiological studies (63.2%). The majority (55.2%) said that are unaware of legal data protection rules in their countries.

**Conclusions:**

IM apps are used by medical professionals worldwide to share and discuss clinical data and are preferred to many other methods of data sharing and are often used to share many different types of clinical data. They are perceived as a fast and easy way of communication, but medical professionals should be aware of the appropriate use of IM to prevent legal and privacy issues.

**What’s new?**

- A total of 125 responders from 13 Latin-American countries (79% male, mean age 46.1 ± 9.7 years) completed the survey, which consisted in 22 questions, in Spanish and Portuguese.
- Most of the responders declared that they send (88.8%) and receive (97.6%) clinical data through IM apps.
- Most senders declare that they anonymize clinical data before sending (71.0 ± 38.3%), but that the data received is anonymized only in 51.4 ± 33.5%.
- The most common tests shared with other physicians were 12-lead electrocardiograms (99.2%), followed by Holter recordings (68.0%) and tracings from electrophysiological studies (63.2%).
- The majority (55.2%) said that are unaware of legal data protection rules in their countries.

## Introduction

Telemedicine consists of providing medical care using communication technologies. It is nowadays a relevant component of contemporary care. Its use has increased exponentially in the context of the coronavirus disease 2019 pandemic caused by severe acute respiratory syndrome coronavirus 2.^1–3^

The widespread availability of smartphones all over the world has been a potent catalyst of telemedicine. Instant messaging (IM) platforms are a prominent component of telemedicine.^4^ However, its use is not yet well regulated or controlled, at least in most of the countries where it is used. Many forms of misuse have been observed, both by medical professionals, but also among patients and physicians. The traditional patient-physician relationship has been dramatically changed with the advent of these IM platforms, sometimes for good, sometimes not.

There is increasing concern in the various health actors in the preservation of patient’s rights. This is due to the advance in medical bioethics and also to the concern of institutions that their personnel adhere to legal practices. Two of the most important rights are medical secrecy and the confidentiality of clinical information. There is even a need for the express consent of the patients to be able to use their information anonymously, in a way that cannot be associated with the patient.

The objective of the study was to inquire about the use of IM, the platforms used, frequency, recipients, contents, the perceived advantages and disadvantages, and protection of personal data by users and institutions in Latin America.

## Methods

A descriptive cross-sectional observational study was done, using a closed questions survey, through an online commercial platform (SurveyMonkey). The form was based on the European Heart Rhythm Association (EHRA) e-Communication and the Scientific Initiative Committees online questionnaire already published.^5^

Questions were made on an individual basis and collected anonymously, but age, gender and country of origin were recorded. Participants could leave responses blank, except country of origin. If less than 50% of the questionnaire was answered, that survey was not included in the analysis.

### Population

Cardiologists working in Latin America in Electrophysiology Services, in Clinical Arrhythmia Departments or Intensive Care Units were included, regardless of whether they perform invasive electrophysiological procedures (IEPs) or the degree of training. If they performed any type of IEP they were considered electrophysiological cardiologists, if they did not perform IEP but worked in a critical care unit they were considered intensive care cardiologists, if they did not perform IEP or assist patients in critical units they were considered clinical arrhythmia cardiologists.

The call was made through the mailing list of active members of the Latin American Heart Rhythm Society (LAHRS). LAHRS has active partners in all countries of the region. The call was also made through local lists of members. LAHRS membership was not required to participate. Its participation and completion was not mandatory nor was encouraged by any economic and/or material good. The same survey had a version in Portuguese and another in Spanish that were merged later. It was available to be answered between 20 November 2020 and 4 January 2021.

The survey consisted of 22 questions in three blocks (see Supplementary material online):

- The first block consisted of seven questions regarding personal information including age, gender, working environment, working position, and experience.
- The second block (11 questions) was about the use of IM, asking the physician if she/he was using IM, which app, frequency of use, anonymization of data, type of data shared, pros and cons about IM.
- The last four questions were related to regulations and protection of data.

The IM platforms included were; cell phone text messages, WhatsApp®, Facebook Messanger®, Linkedln Direct Message®, Microsoft teams®, and Twitter®. The latter is a social media platform, but it was included because it also allows you to send personal messages and is a medium where medical cases are often shared.

### Statistical analysis

The analysis was performed with Microsoft® Excel® 2016 MSO. The quantitative variables are expressed as mean and standard deviation and the qualitative variables in absolute number and percentages. Comparative analyses were not performed.

## Results

**Figure 1:**
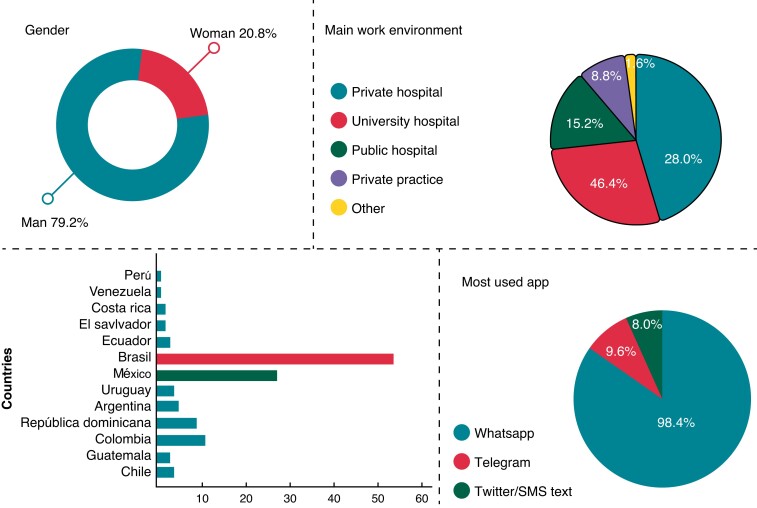
Demographic characteristics of responders to the LAHRS survey. LAHRS, Latin American Heart Rhythm Society.

A total of 125 responders from 13 Latin-American countries (79% male, mean age 46,1 ± 9,7 years) completed the survey. Details are shown in *Table 1* and *Figure 1*.

**Table 1:** General characteristics of the responders of the LAHRS survey on instant messaging

**Main work environment**	*n* (%)
ȃPrivate hospital	58 (46.4)
ȃUniversity hospital	35 (28.0)
ȃPublic hospital	19 (15.2)
ȃOther	13 (10.4)
**Current working position**	*n* (%)
ȃHead of staff	48 (38.4)
ȃStaff physician	72 (57.6)
ȃFellow	1 (0.8)
ȃOther	4 (3.2)
**Main medical specialty**	*n* (%)
ȃCardiology, electrophysiology	97 (77.6)
ȃCardiology, clinical arrhythmia	23 (18.4)
ȃCardiology, intensive care	3 (2.4)
ȃOther	2 (1.6)

**Figure 2:**
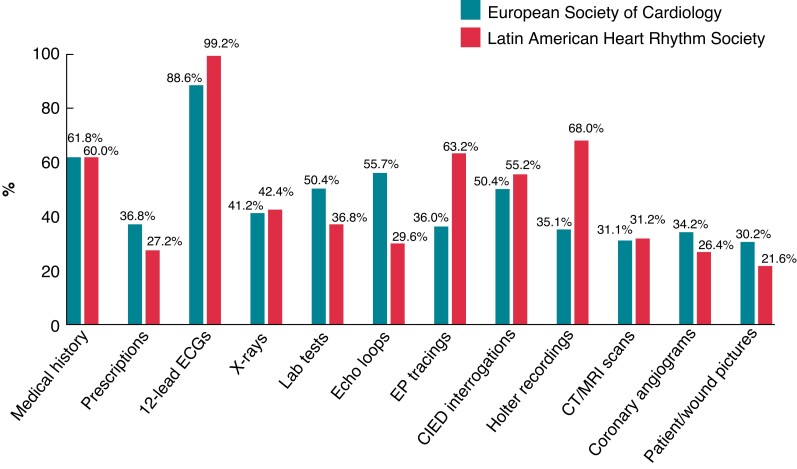
Characteristics of received and sent clinical data through IM tools. IM, Instant messaging.

Most of the responders declared that they send (88.8%) and receive (97.6%) clinical data through IM apps. They reported that clinical data was shared with cardiologists from their own institution in 82.4% or with colleagues from other institutions in 67.2%; they also shared clinical data with fellows in training in 55.2%. Most of them use the apps for discussion of cases more than one time a day (46.4%), or at least once in a week in 40%. The most common used app was WhatsApp (98.4%), followed by Telegram (9.6%), Twitter or ordinary SMS text (8.0%) (*Figure 2*).

Most senders declare that they anonymize clinical data before sending (71.0 ± 38.3%), but that the data received is anonymized only in 51.4 ± 33.5%. The most common tests shared with other physicians were 12-lead electrocardiograms (ECGs) (99.2%), followed by Holter recordings (68.0%) and tracings from electrophysiological studies (63.2%).

**Figure 3:**
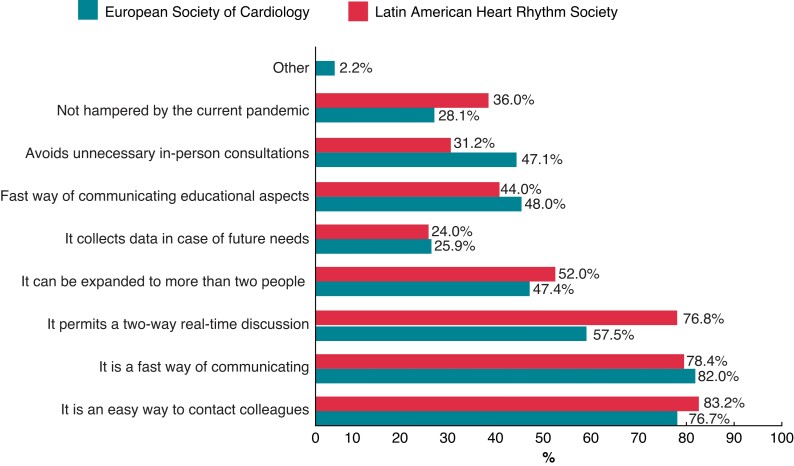
Advantages of instant messaging in sharing clinical data, as pointed by LAHRS survey responders, compared to the ones from the EHRA survey.^5^ LAHRS, Latin American Heart Rhythm Society.

The responders of the LAHRS survey pointed some advantages of using IM in sharing clinical data, and those are listed in *Figure 3*. The main reason to use IM apps to share data was the perception of a simple (83.2%) and fast (78.4%) way to get into contact with colleagues, followed by the ability to discuss in real-time (76.8%).

**Figure 4:**
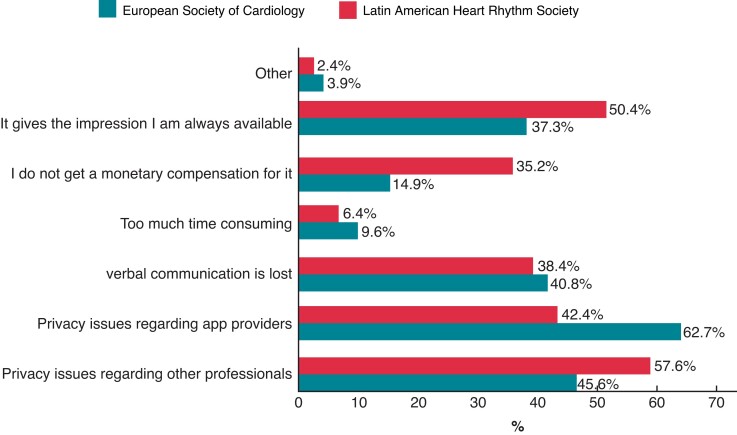
Disadvantages of instant messaging in sharing clinical data, as pointed by LAHRS survey responders, compared to the ones from the EHRA survey.^5^ EHRA, European Heart Rhythm Association; LAHRS, Latin American Heart Rhythm Society.

Most of the responders see some disadvantages, as shown on *Figure 4*, like problems with privacy from their colleagues (57.6%) or apps providers (42.4%), as well as feeling that one should be available all the time (50.4%).

The responders stated that use IM to contact directly with patients (73.6%), and the majority (55.2%) said that are unaware of legal data protection rules in their countries. The vast majority (90.4%) use mobile phone security strategies like fingerprint or face recognition to prevent that other can have access to data from electronic devices.

## Discussion

IM is routinely used by medical professionals in Latin-America. The most frequent aim of this communication is discussing clinical data, ECG and Holter recordings. This type of communication is perceived as a simple (83.2%) and fast (78.4%). Most of senders declare that they anonymize clinical data before sending (71%), but that the data received is less frequently anonymized (51%). The majority (55%) said that are unaware of legal data protection rules in their countries, even though the vast majority (90%) use security strategies to prevent privacy violation of the data from electronic devices.

The vast majority of cardiologists who work in Latin American electrophysiology services and participate in the survey use new digital tools for communication in their medical practice, mostly smartphone-based IM apps. They mostly use them to share results of different types of tests, like ECG, Holter recordings and tracings from electrophysiological studies. Although the majority referred to anonymize the clinical data before sharing, most of the responders stated that they receive data without this kind of caution. This inconsistency should be further investigated. The majority of responders claimed to be unaware of legal data protection rules in their countries.

Findings in this survey are similar to the ones reported in the EHRA-SMS survey.^5^ New IM tools increase the possibilities of interaction with colleagues, in real time and even physically separated. But the use of IM as a communication tool has its pitfalls too, as many of the responders feel that the personal availability all time maybe a disadvantage. The balance between these situations should be done in a way to improve patient care.

There were a few differences between responders in our survey and the one from Europe. The responders in the Latin-America are a little older (mean age of 46 years, as compared with 43 in the European one), and there are important differences in the working environment (main work in private hospitals in Latin-America, rather than Public Hospitals in Europe), that reflects the health care system differences in between our regions. Another important difference is the low number of Fellows that responded the Survey in our region (less than 1%), in contrast with 20% observed in the European one. This may be due to the fewer access to specialized social media by training physicians in our region, as younger colleagues undoubtedly have access to smartphones and IM tools. Indeed, more specialists (more than 77%) responded the Latin-American survey, as compared to 58% in the European one. This fact could be due to selection bias.

Most responders declare that they anonymize clinical data before sending (71.0 ± 38.3%), but that the data received is anonymized only in 51.4 ± 33.5%. This was very different than the observation in the European survey,^5^ as the responders said that data was anonymized on 57% in shared and 56% when received, although this difference may be due to a ‘socially desirable responding’ bias that could be detected in our survey, but not on the European one. The use of IM and social media in professional settings will certainly grow further in the near future.^6^

## Conclusions

IM is routinely used by medical professionals in Latin-America. The most frequent aim of this communication is discussing clinical data, ECG, and Holter recordings. Most of senders declare that they anonymize clinical data before sending (71%). A great proportion (55%) are unaware of legal data protection rules in their countries.

### Limitations

The main limitation of this study is that the sample of physicians surveyed was not taken randomly, nor by any other method that could guarantee the representativeness of the universe studied. This also limits the external validity of any subgroup comparison that might be considered.

## Supplementary material

Supplementary material is available at *Europace* online.

## Supplementary Material

euac080_Supplementary_Data

## Data Availability

The data underlying this article will be shared on reasonable request to the corresponding author.
