# Supplementary material for: Use of instant messaging in electrophysiological clinical practice in Latin America: a LAHRS survey
Source: Europace. 2022 Jun 21;25(1):59–64. doi: 10.1093/europace/euac080 (PMC10103556; doi:10.1093/europace/euac080)
Supplement: euac080_Supplementary_Data [file euac080_supplementary_data.docx]

**Supplementary material 1: Questionnaire**

1. Age

1.1. Years (values from 20-100)

…………………………………………………………………………………………………………………………………………………

2. Sex

2.1. Man

2.2. Woman

…………………………………………………………………………………………………………………………………………………

3. In which country is your center located?

3.1. Choice one option (“all Latin American countries”)

…………………………………………………………………………………………………………………………………………………

4. What is your main working environment?

4.1. University Hospital

4.2. Public hospital

4.3. Private hospital

4.4. private practice

4.7. Other: specify (“free text”)

…………………………………………………………………………………………………………………………………………………

5. What is your job title at your current main job?

5.1. Head of Service, Head of Department or higher position

5.2. Associated Physician

5.3. Resident or Fellow

5.4. Other: specify (“free text”)

…………………………………………………………………………………………………………………………………………………

6. What is your main job specialty?

6.1. Clinical cardiology / arrhythmology

6.2. Cardiology / invasive electrophysiology (perform procedures)

6.3. Cardiology at cardiac intensive care unit

6.4. Other: specify (“free text”)

…………………………………………………………………………………………………………………………………………………

7. How much do you like each of the following methods of contacting your colleagues to discuss clinical data?

(Lickert´s scale: 1; not at all, 2; not much, 3; indifferent, 4; good, 5; very much)

7.1. Live contact (inpatient, outpatient)

7.2. Phone call

7.3. Video call

7.4. Email

7.5. Instant messaging

…………………………………………………………………………………………………………………………………………………

8. Do you send clinical data for discussion to your colleagues through instant messaging applications (SMS, WhatsApp, Telegram, etc.)?

8.1. Yes

8.2. No

…………………………………………………………………………………………………………………………………………………

9. Do you receive clinical information sent to you by a colleague for clinical discussion via instant messaging?

9.1. Yes

9.2. No

…………………………………………………………………………………………………………………………………………………

10. With whom do you usually share clinical data?

(“check all that apply”)

10.1. Students from my department or institution

10.2. Cardiologists from my department or institution

10.3. Cardiologists from other institutions

10.4. Partners/colleagues from other specialties at your institution

10.5. Partners/colleagues from other specialties from other institutions

10.6. General practitioners

10.7. Nurses / paramedics / technicians

10.8. Administrative staff

…………………………………………………………………………………………………………………………………………………

11. How often do you use instant messaging to share or discuss clinical data?

11.1. Less than once a month

11.2. At least once a month

11.3. At least once a week

11.4. At least once a day

11.5. More than once a day

…………………………………………………………………………………………………………………………………………………

12. What instant messaging services do you use to share or discuss clinical data?

(“check all that apply”)

12.1. SMS (Cell Phone Native Text Message)

12.2. WhatsApp

12.3. Telegram

12.4. Facebook Messenger

12.5. Twitter message

12.6. LinkedIn Direct Message

12.7. Microsoft teams

12.8. Other: specify (“free text”)

…………………………………………………………………………………………………………………………………………………

13. How often do you anonymize clinical data before sharing it via instant messaging?

13.1. Percentage: (0% - Never; 100% all the time)

…………………………………………………………………………………………………………………………………………………

14. How often is the clinical data you receive non-anonymous?

14.1. Percentage: (0% - Never; 100% all the time)

…………………………………………………………………………………………………………………………………………………

15. What kind of clinical data do you share or discuss via instant messaging?

(“check all that apply”)

15.1. Medical history

15.2. Prescriptions

15.3. 12-leads ECG

15.4. X-rays images

15.5. Lab tests

15.6. Echocardiogram images

15.7. EP traces

15.8. Pacemaker / ICD / Resynchronizer Interrogations

15.9. Short and long term Holter recordings (includes loop monitor)

15.10. CT scans/MRIs

15.11. Coronary angiograms

15.12. Patient/wound images

…………………………………………………………………………………………………………………………………………………

16. What do you think are the advantages of sharing or discussing clinical data with your colleagues via instant messaging? (“check all that apply”)

16.1. It is a simple way to contact my colleagues/colleagues

16.2. It's a quick way to communicate

16.3. Enables two-way discussion in real time

16.4. It can be extended to more than two people.

16.5. Collect data in case of future needs

16.6. It constitutes a fast track for educational aspects (training cases)

16.7. Avoid unnecessary face-to-face consultations and moving patients

16.8. One is not hampered by the current pandemic

16.9. Other (blank)

…………………………………………………………………………………………………………………………………………………

17. What do you think are the disadvantages of sharing or discussing clinical data with your colleagues via instant messaging? (“check all that apply”)

17.1. Privacy issues regarding sharing with colleagues not really involved in the case

17.2. Privacy issues regarding App providers

(Where is data stored? Can it be sold or reused?)

17.3. Non-verbal communication is lost.

17.4. Too much time.

17.5. I get no monetary compensation for it.

17.6. I always have to be available

17.7. Other

…………………………………………………………………………………………………………………………………………………

18. Do you use instant messaging applications to share/discuss clinical data with your patients?

18.1 Yes, all the time.

18.2 Yes, only sometimes.

18.3 No, never.

19. Are you aware of the general data protection regulation on data protection and privacy?

19.1. Yes

19.2. No / I didn´t know it existence in my country

…………………………………………………………………………………………………………………………………………………

20. Does your institution have specific policies/regulations in terms of instant messaging for professional use?

20.1. Yes

20.2. No

20.3. I don´t know

…………………………………………………………………………………………………………………………………………………

21. Do you protect your mobile phone and PC by passcode/face scan/fingerprint or similar methods?

21.1. Yes

21.2. No

…………………………………………………………………………………………………………………………………………………

22. Does your institution provide you with a legally approved instant messaging service for professional use?

22.1. Yes

22.2. No

22.3. I don´t know

…………………………………………………………………………………………………………………………………………………
